# Description of two new *Stenohya* species from China (Pseudoscorpiones, Neobisiidae)

**DOI:** 10.3897/zookeys.213.2237

**Published:** 2012-08-01

**Authors:** Jun-Fang Hu, Feng Zhang

**Affiliations:** 1 College of Life Sciences, Hebei University, Baoding 071002, China

**Keywords:** Pseudoscorpions, Neobisiidae, *Stenohya*, new species, China

## Abstract

Two new species of the genus *Stenohya* Beier, 1967 are described from China: *Stenohya pengae***sp. n.** (male and female; type locality Daming Mountain, Nanning City, Guangxi Zhuang Autonomous Region) and *Stenohya huangi* **sp. n.** (female; type locality Gushan Mountain, Fuzhou City, Fujian Prov.).The presence of *Stenohya pengae* **sp. n.** in the tree crown of *Castanopsis fabri* represents a new habitat for Neobisiidae. A key and a distribution map of the Chinese *Stenohya* species are also provided.

## Introduction

*Stenohya* Beier, 1967 is a small Asian pseudoscorpion genus of the family Neobisiidae Chamberlin, 1930. At present it includes 12 species (Harvey 2011; Zhao et al. 2011), of which three have been reported from China: *Stenohya chinacavernicola* Schawaller, 1995 (Sichuan Province), *Stenohya curvata* Zhao et al., 2011 (Yunnan Province) and *Stenohya xiningensis* Zhao et al., 2011 (Qinghai Province).

Damingshan National Nature Reserve is located in the midwest of Guangxi Zhuang Autonomous Region, lying on the Tropic of Cancer, and possesses a rich subtropical primeval forest, which is home to many rare animals and plants. Daming Mountains are densely covered by jungle, including trees of the families Fagaceae, Styracaceae, Daphniphyllaceae, Lauraceae and Ericaceae (Ding 1991). In 2011 we collected some *Stenohya* specimens living on the leaves of the tree *Castanopsis fabri* Hance, 1884 (Fagaceae) by sweeping vegetation with an entomological net. After examining the specimens in the laboratory, we found them to represent a new species, which is described here under the name *Stenohya pengae* sp. n. When we examined the pseudoscorpions collected by Prof. Fusheng Huang from Gushan Mountain, Fujian Province, China, we found another new *Stenohya* species, which is also described and illustrated in this paper as *Stenohya huangi* sp. n.

## Material and methods

The specimens are preserved in 75% alcohol and deposited in the Museum of Hebei University (MHBU). Permanent slide mounts were prepared by removing the chelicerae, pedipalps, leg I and leg IV from specimens with small needles and clearing overnight with lactic acid at room temperature. Drawings were made with the aid of a camera lucida mounted above the eyepiece of a compound microscope. Photographs were taken with a Leica M165 stereomicroscope. Terminology of trichobothria follows Chamberlin (1931). The term “rallum” (for flagellum) is adopted following Judson (2007). The following abbreviations are used in the text for the trichobothria: *b* = basal; *sb* = sub-basal; *st* = sub-terminal; *t* = terminal; *ib* = interior basal; *isb* = interior sub-basal; *ist* = interior sub-terminal; *it* = interior terminal; *eb* = exterior basal; *esb* = exterior sub-basal; *est* = exterior sub-terminal; *et* = exterior terminal.

### Key to the Chinese species of the genus *Stenohya*

**Table:** 

1	Cave-living species, with single pair of eyes reduced to spots	*Stenohya chinacavernicola* Schawaller, 1995
–	Free-living species, with two pairs of eyes, anterior pair with lens and posterior pair represented by eyespots or weak lenses	2
2	Trichobothria *it* and *et* at same level	*Stenohya huangi* sp. n.
–	Trichobothrium *it* posterior to *et*, situated midway between *est* and *et*	3
3	Male with slender pedipalps (femur 6.79–7.20, patella 6.17–6.25 times longer than broad)	*Stenohya pengae* sp. n.
–	Male with less slender pedipalps (femur 5.00–6.42, patella 3.29–4.68 times longer than broad)	4
4	Movable chelal finger with more than 70 contiguous teeth; male sternites V–X with a pair of medial discal setae; male chela with movable chelal finger straight, hand without a spine	*Stenohya xiningensis* Zhao et al., 2011
–	Movable chelal finger with less than 50 teeth, which are not contiguous; male sternites VI–VIII with a pair of medial discal setae; male chela with movable chelal finger curving in basal third in ventral view, hand with a spine	*Stenohya curvata* Zhao et al., 2011

#### Stenohya pengae sp. n.

urn:lsid:zoobank.org:act:3E8D205C-B127-4BE0-AC87-B029B7F9719F

http://species-id.net/wiki/Stenohya_pengae

Figs 1 –8 10–18

##### Type material.

Holotype male (Ps.-MHBU-GX110521), China: Guangxi Province, Nanning City, Daming Mountain [23°08'N, 108°17'E], alt. 1250 m, 21 May 2011, Yan-qiu Peng leg. Tree-crown layer of *Castanopsis fabri*. Paratypes: 17 males and 25 females, same data as for holotype.

##### Etymology.

The specific name is a patronym in honour of Ms Yan-qiu Peng, who collected the specimens.

##### Diagnosis.

Movable cheliceral finger with one seta; movable chelal finger with 45–47 teeth; male pedipalpal chela 4.58–4.64 (female 4.09–4.25) times longer than broad; trichobothrium *it* halfway between *est* and *et*.

##### Description of male

**Figure 1:**
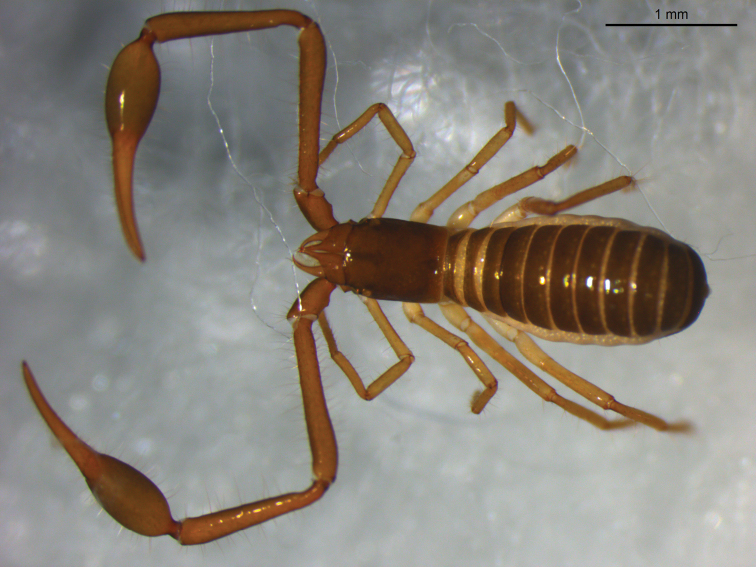
*Stenohya pengae* sp. n., dorsal view of male holotype.

(Fig. 1). Colour mostly dark brown, pedipalps and legs reddish brown. Setae of body straight and acicular.

**Figures 2–9.**
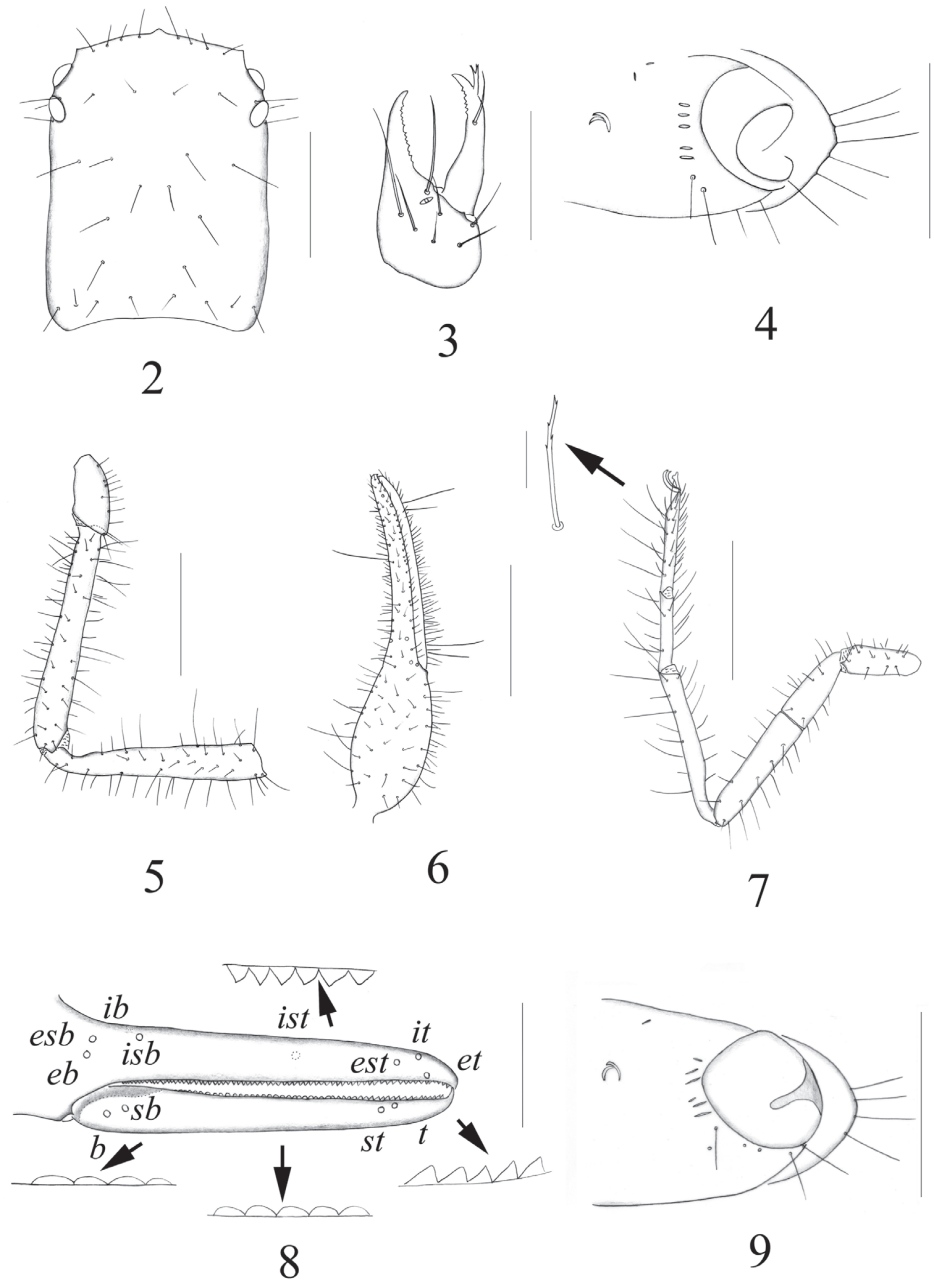
*Stenohya pengae* sp. n., male holotype (**2–7**) and *Stenohya xiningensis* Zhao et al., 2011,male (**9**) **2** Carapace, dorsal view **3** Right chelicera **4** Coxa of right pedipalp, lateral view, showing lyrifissures **5** Pedipalp (minus chela), dorsal view **6** Chela, dorsal view **7** Leg IV **8** Fingers of right chela, lateral view, showing trichobothriotaxy and teeth; *Stenohya xiningensis* **9** Coxa of right pedipalp, lateral view, showing lyrifissures. Scale bars: 1 mm (**5–7**); 0.5 mm (**2, 4, 8–9**); 0.4 mm (**3**).

Carapace (Fig. 2) smooth, longer than broad, with a total of 34–36 setae, including 8 on anterior margin and 8 on posterior margin; paired lyrifissures near the eyes and posterior margin; epistome small and triangular; 4 eyes, anterior pair with well developed lens, posterior pair with weak lens.

Abdomen. Pleural membrane granulate. Tergal chaetotaxy: 6: 8: 8–10: 10–12: 10–11: 11–12: 11–12: 10–11: 10–11: 9–11: 6–8: 2, including at least 4 tactile setae on tergites V–XI. Anterior genital operculum (Fig. 16) with 23–24 setae; posterior genital sternite with 12–14 scattered setae and 2 lyrifissures;. chaetotaxy of remaining sternites (IV–XI) 20–22: 22–24: 22–24: 20–24: 18–20: 19–22: 15–18: 8–10: 2, sternites VI–VIII (Fig. 14) with 13–15 medial discal setae.

**Figures 10–18.**
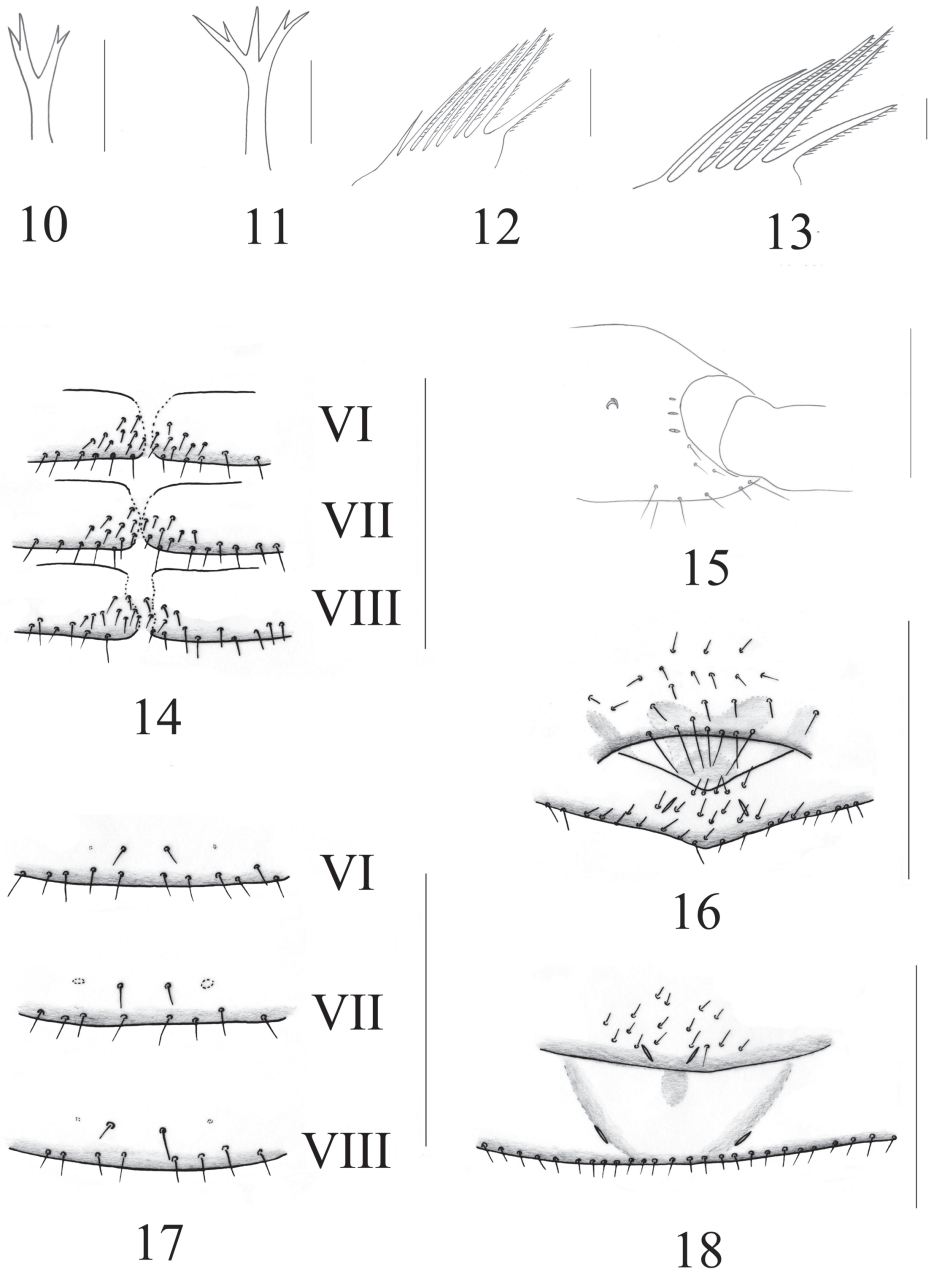
*Stenohys pengae* sp. n. **10** Galea, male **11** Galea, female **12** Rallum, female **13** Rallum, male **14** Male sternites VI–VIII, with 13–15 medial discal setae **15** Coxa of right pedipalp, female, lateral view, showing lyrifissures **16** Genital sternites, male **17** Female sternites VI–VIII with 2 medial discal setae **18** Genital sternites, female. Scale bars: 1 mm (**14–15**); 0.5 mm (**16–18**); 0.05 mm (**10–13**).

Pedipalps (Fig. 4) smooth; apex of coxa rounded and with 5 setae; lateral face of coxa with 3–5 ordinary lyrifissures near margin of foramen, plus 1–3 dorsally and 2 curved posterior maxillary lyrifissures. Venom apparatus present only in fixed chelal finger, venom duct very short. Femur straight; patella claviform and internal face with a tubercle at base. Trichobothrial pattern (Fig. 8): *eb* and *esb* situated near base of finger, grouped very closely with *ib* and *isb*; *est*, *et* and *it* grouped together near finger tip; *ist* nearer to *it* than to *isb*; *b* and *sb* situated closer to each other in basal half, *st* and *t* close to each other in distal half of movable finger. Fixed chelal finger with 70–74 pointed teeth of unequal length, movable finger with about 45–47 teeth, which are pointed and of slightly unequal length in distal part, rounded in middle part and low in basal part, all teeth contiguous.

Chelicera (Fig. 3). Palm with 7 setae (only one male with 7 on left and 8 on right cheliceral palm), movable finger with 1 sub-medial seta; fixed finger with 13–14 teeth; movable finger with 6–7 teeth; serrula exterior with 40–43 lamellae; serrula interior with 30–32 lamellae; galea (Fig. 10) elongated and divided into two main branches, each branch secondarily divided into 2 terminal branchlets; rallum (Fig. 13) of 6 blades, all blades with anteriorly-directed spinules except the basal-most blade, distalmost blade somewhat widened at its base and distinctly shorter than second blade.

Leg IV (Fig. 7). Tibia with 4 tactile setae (TS 0.14–0.26, 0.30–0.48, 0.62–0.74 and 0.86–0.96), basitarsus with 4 tactile setae (TS 0.13–0.15, 0.25–0.42, 0.62–0.67 and 0.84–0.87), telotarsus with 3 tactile setae (TS 0.10–0.14, 0.27–0.36 and 0.51–0.63). Subterminal setae bifurcate, dorsal branch also terminally bifurcate; arolium not divided, shorter than claws, which are slender and simple.

**Dimensions (in mm) and ratios (in parentheses).**Body length 3.3–3.6. Carapace 1.05–1.10/0.82–0.85 (1.28–1.29); diameter of anterior eye 0.10–0.11; diameter of posterior eye 0.10–0.12. Pedipalps: trochanter 0.60–0.70/0.25–0.32 (2.19–2.40), femur 1.80–1.90/0.25–0.28 (6.79–7.20), patella 1.75–1.85/0.28–0.30 (6.17–6.25), chela (with pedicel) 2.38–2.55/0.52–0.55 (4.58–4.64), chela (without pedicel) 2.18–2.40 (4.19–4.36), hand length (without pedicel) 0.95–1.05 (1.83–1.91), movable finger length 1.35–1.40 (1.33–1.42 times longer than hand without pedicel). Chelicera 0.55–0.60/0.30–0.32 (1.83–1.88), movable finger length 0.35–0.44. Leg I: femur 0.85–0.87/0.14–0.18 (4.83–6.07), patella 0.55–0.58/0.13–0.15 (3.87–4.23), tibia 0.60–0.65/0.10–0.11 (5.91–6.00), basitarsus 0.45–0.48/0.09–0.10 (4.80–5.00), telotarsus 0.50–0.52/0.09–0.10 (5.20–5.56). Leg IV: femur + patella 1.45–1.55/0.22–0.24 (6.46–6.59), tibia 1.10–1.15/0.13–0.15 (7.67–8.46), basitarsus 0.55–0.58/0.08–0.10 (5.80–6.88), telotarsus 0.65–0.70/0.08–0.10 (7.00–8.13).

##### Description of female.

Like male, except as follows. Carapace with a total of 30–34 setae, including 6 setae on anterior margin and 6–8 setae on posterior margin. Tergal chaetotaxy: 6–10: 8–10: 9–10: 10–12: 11–12: 10–12: 11–13: 12–15: 11–13: 11–13: 6–8: 2, including at least 4 tactile setae on tergites IV–XI. Anterior genital sternite (Fig. 18) with 16–18 small marginal setae and 2 lyrifissures; posterior genital sternite with 28–32 marginal setae and 2 lyrifissures; chaetotaxy of remaining sternites (IV–XI) 24–30: 23–27: 22–26: 19–22: 20–21: 17–20: 15–19: 7–8: 2, sternites VI–VIII (Fig. 17) with a pair of medial discal setae, clearly longer than marginal setae.

Pedipalps. Lateral face of coxa with 3–5 ordinary lyrifissures at margin of foramen, plus 0–3 at dorsal margin and 2 curved lyrifissures; fixed chelal finger with 66–79 teeth, movable finger with about 45–55 contiguous teeth which are pointed and of slightly unequal length in distal half, rounded and low in basal half.

Chelicera. Palm with 7 setae (two females with 8 on left and 7 on right cheliceral palm, one female with 6 on left and 7 on right palm), movable finger with 1 sub-medial seta; serrula exterior with 34–36 lamellae; serrula interior with 28–33 lamellae; galea (Fig. 11) elongate and divided into three main branches, two of which are secondarily divided into 2 terminal branchlets; rallum (Fig. 12) of 8 blades.

**Dimensions (in mm) and ratios.** Body length ca. 3.9–5.0. Carapace 0.95–1.00/0.80–0.85 (1.15–1.28); diameter of anterior eye 0.11–0.13; diameter of posterior eye 0.12–0.14. Pedipalps: trochanter 0.53–0.55/0.25–0.30 (1.83–2.12), femur 1.40–1.45/0.24–0.28 (5.18–5.83), patella 1.10–1.15/0.28–0.30 (3.83–3.93), chela (with pedicel) 2.25–2.33/0.53–0.57 (4.09–4.25), chela (without pedicel) 2.10–2.18 (3.82–3.96), hand length (without pedicel) 0.85–0.95 (1.60–1.67), movable finger length 1.17–1.20 (1.26–1.38 times longer than hand without pedicel). Chelicera 0.70–0.75/0.35–0.40 (1.88–2.00), movable finger length 0.45–0.50. Leg I: femur 0.70–0.80/0.13–0.14 (5.38–5.71), patella 0.45–0.50/0.13–0.14 (3.46–3.57), tibia 0.50–0.55/0.09–0.10 (5.50–5.56), basitarsus 0.35–0.37/0.08–0.09 (4.11–4.38), telotarsus 0.43–0.45/0.09–0.10 (4.50–4.78). Leg IV: femur + patella 1.30–1.40/0.23–0.24 (5.65–5.83), tibia 1.10–1.15/0.13–0.14 (8.21–8.46), basitarsus 0.50–0.55/0.08–0.10 (5.50–6.25), telotarsus 0.60–0.65/0.08–0.10 (6.50–7.50).

##### Distribution.

This species is known only from the type locality.

##### Remarks.

Three *Stenohya* species have been previously recorded from China: *Stenohya chinacavernicola* Schawaller, 1995, *Stenohya curvata* Zhao et al., 2011 and *Stenohya xiningensis* Zhao et al., 2011. *Stenohya pengae* sp. n. can easily be separated from these species by its extremely slender pedipalpal segments, 4 well-developed eyes, the absence of a spine at the base of the male chelal hand, and the presence of medial discal setae on male sternites VI–VIII only.

The new species resembles *Stenohya martensi* (Schawaller, 1987) in having slender pedipalps, but it can be distinguished from the latter by the movable cheliceral finger having only one seta (two in *Stenohya martensi*), the movable chelal finger with 45–47 teeth (more than 80 in *Stenohya martensi*) and the male pedipalpal chela 4.58–4.64 times longer than broad (6.2 times in *Stenohya martensi*). *Stenohya caelata* (Callaini, 1990) and *Stenohya kashmirensis* (Schawaller, 1988) differ from *Stenohya pengae* sp. n. in having granules on the pedipalpal femur and patella, and the cheliceral palm with 5 or 6 setae. The new species can be easily distinguished from *Stenohya mahnerti* Schawaller, 1994, *Stenohya hamata* (Leclerc and Mahnert, 1988) and *Stenohya gruberi* (Ćurčić, 1983) by the more slender pedipalpal femur and patella. *Stenohya heros* (Beier, 1943) has less slender pedipalp in female (femur 4.5 vs. 5.18–5.83 times, patella 3.2 vs. 3.83–3.93, chela (with pedicel) 3.3 vs. 4.09–4.25, movable finger 1.0 vs. 1.26–1.38 times longer than hand without pedicel). *Stenohya vietnamensis* Beier, 1967 and *Stenohya lindbergi* (Beier, 1959) were both described from nymphs, but *Stenohya vietnamensis* lacks an epistome and *Stenohya lindbergi* has more teeth (78) on the movable chelal finger.

Specimens of *Stenohya pengae* were found on the leaves of *Castanopsis fabri*, which represents an exceptional habitat for Neobisiidae. Neobisiidae generally live in leaf litter and soil, under rock, bark and in caves, although they have sometimes been found climbing young trees and shrubs (Weygoldt, 1969). Fourty-one specimens of *Stenohya pengae* were collected by sweeping trees of *Castanopsis fabri* with an entomological net; only two were found on stone steps and these might have been dislodged from the trees. The collector also examined the tree bark and leaf litter around the trees, without finding any specimens of *Stenohya pengae*.

Approximately 100 pseudoscorpion specimens were collected from Fujian and Guangdong provinces were extracted by beating shrubs, of which 74 (including 4 protonymphs, 2 deutonymphs, 11 tritonymphs and 57 adults) belong to the genus *Geogarypus* Chamberlin, 1930 (family Geogarypidae Chamberlin, 1930) and 22 tritonymphs belong to the genus *Bisetocreagris* Ćurčić, 1983 (family Neobisiidae). All of the *Stenohya pengae* specimens were adults. Adis and Mahnert et al. (1988) found that *Brazilatemnus browni* Muchmore was bivoltine, with one generation occuring in the trunk/canopy habitat in April/May (during forest inundation) and the second in the forest floor in November/December (non-inundation period). It might therefore be interesting to look for *Stenohya pengae* in both habitats at different times of the year.

#### Stenohya huangi

sp. n.

urn:lsid:zoobank.org:act:AED176C9-840E-4C15-9981-8777D01C6632

http://species-id.net/wiki/Stenohya_huangi

Figs 19 –28

##### Type material.

Holotype female (Ps.-MHBU-FJ750224), China: Fujian Province, Fuzhou City, Gushan Mountain [26°04'N, 119°21'E], 24 February 1975, Fu-sheng Huang. Habitat unknown.

##### Etymology.

The specific name is a patronym in honour of Prof. Fu-Sheng Huang, who collected and donated the specimen.

##### Diagnosis.

Species with slender pedipalps (femur 6.40, patella 5.25, chela with pedicel 4.87, chela without pedicel 4.57 times as long as broad) and slender legs IV (e.g. femur+patella 7.23 times as long as deep), with low numbers of the teeth (about 30) on movable chelal finger; trichobothria *it* and *et* at same level.

##### Description of female (holotype)

**Figure 19.**
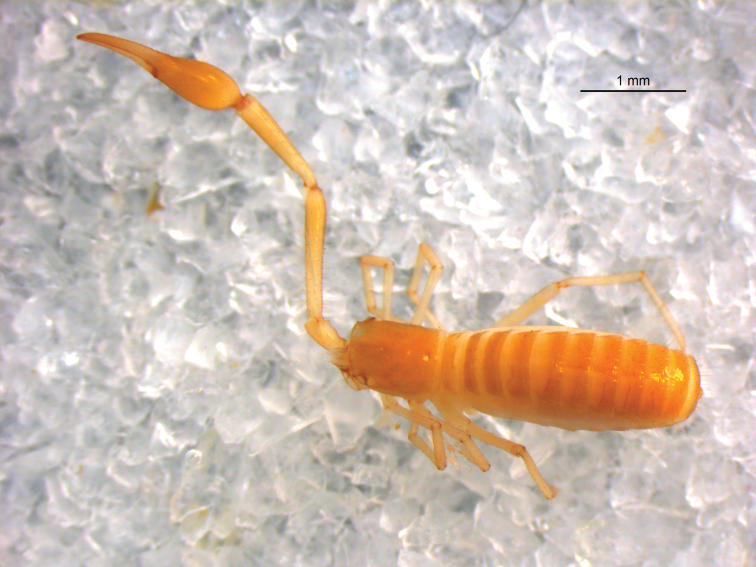
*Stenohya huangi* sp. n., dorsal view of female holotype (chelicerae, left palp and left leg IV removed).

(Fig. 19). Colour mostly yellow, setae of body straight and acicular.

**Figures 20–28.**
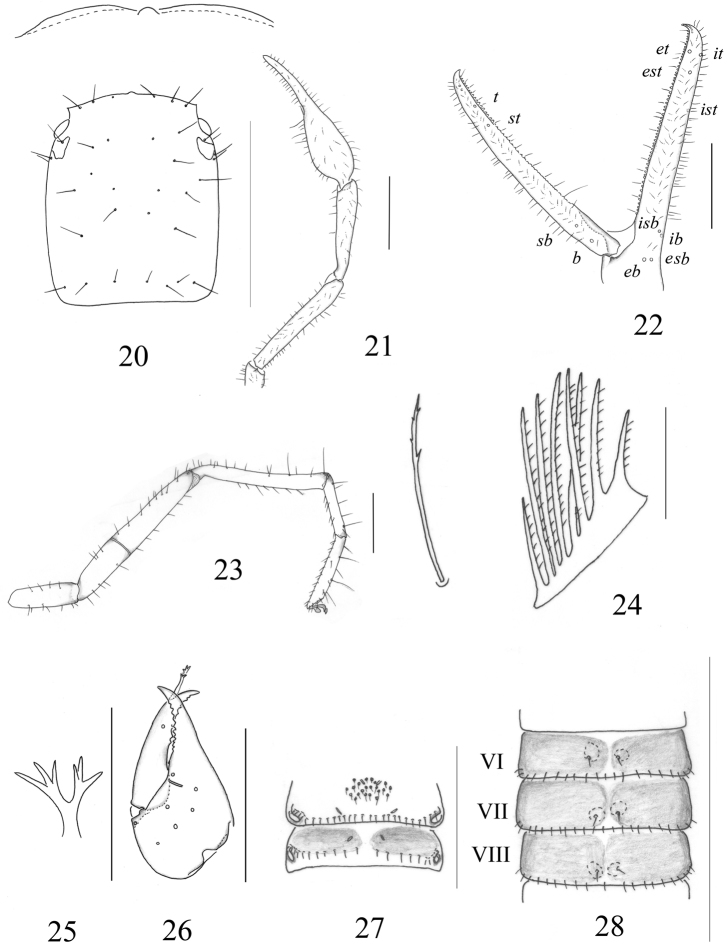
*Stenohya huangi* sp. n., female. **20** Carapace **21** Right pedipalp, dorsal view **22** Fingers of left chela, lateral view **23** Leg IV **24** Rallum **25** Galea **26** Left chelicera **27** Genital sternites **28** sternites VI–VIII, showing paired medial discal setae. Scale bars: 1 mm (**20–21, 28**); 0.5 mm (**22–23, 26–27**); 0.05 mm (**24–25**).

Carapace (Fig. 20) smooth, with a total of 36 setae, including 6 on anterior margin and 8 on posterior margin; epistome small and triangular; 4 eyes, anterior pair with lens, posterior pair with weak lenses; lateral margins slightly convex.

**Figures 29.**
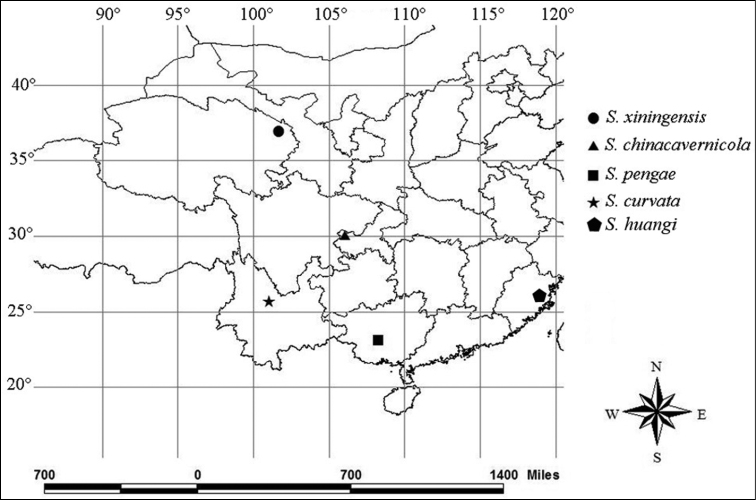
Distribution map of Chinese *Stenohya* species.

Abdomen. Pleural membrane strongly granulate. Tergal chaetotaxy: 4: 12: 10: 10: 10: 10: 11: 11: 11: 10: 9, including at least 4 tactile setae on tergites VI–XI; anterior genital sternite (Fig. 27) with 22 small marginal setae and 2 lyrifissures; posterior genital sternite with 20 marginal setae and 2 lyrifissures; sternal chaetotaxy (IV–XI): 27: 22: 22: 22: 24: 22: 19: 17:10, sternites VI–VIII (Fig. 28) with a pair of discal setae; anal cone with 2 dorsal and 2 ventral setae.

Pedipalps (Figs 21–22). Apex of coxa rounded and with 4 setae, lateral face of coxa with 2 ordinary lyrifissures at margin of foramen, and 2 curved lyrifissures. Anterior face of femur with fine granulation; patella claviform, smooth; chelal fingers long and slender. Trichobothriotaxy: *est*, *et* and *it* grouped together distally; *ist* situated midway between *isb* and *it*, nearer to *it* than to *isb*. *eb* and *esb* situated on base of the hand, grouped very closely with *ib* and *isb*; *b* and *sb* closer to each other situated on the basal half, and *st* and *t* closer to each other situated on the distal half of the movable finger. Fixed chelal finger with 63 pointed teeth of unequal length, movable finger with about 30 teeth which with 20 pointed teeth slightly unequal length in distal half, and 10 rounded teeth in basal half.

Cheliceral palm (Fig. 26) with 7 setae, movable finger with 1 sub-medial seta; fixed finger with 12 teeth; movable finger with 6 teeth; serrula exterior with 30 lamellae; serrula interior with 28 lamellae; galea (Fig. 25) elongated and divided into two main branches, each branch secondarily divided into 3 terminal branchlets; rallum of 8 blades (Fig. 24), all blades with anteriorly-directed spinules, the basalmost blade about half of the length of the others, distalmost blade distinctly shorter than second one and somewhat widened at its base.

Legs (Fig. 23). Tibia IV with 2 tactile setae (TS 0.70, 0.95), basitarsus IV with 2 tactile setae (TS 0.15, 0.81), and telotarsus IV with 2 tactile setae (TS 0.27, 0.61). Subterminal tarsal seta bifurcate; arolium not divided, shorter than slender and simple claws.

**Dimensions (in mm) and ratios (in parentheses).** Body length 4.2. Carapace 1.29/0.89 (1.45); diameter of anterior eye 0.10; diameter of posterior eye 0.09. Pedipalps: trochanter 0.59/0.26 (2.27), femur 1.58/0.25 (6.32), patella 1.38/0.26 (5.31), chela (with pedicel) 2.58/0.53 (4.87), chela (without pedicel) 2.42 (4.57), hand length (without pedicel) 1.04 (1.96), movable finger length 1.44 (1.38 times longer than hand without pedicel). Chelicera 0.67/0.38 (1.76), movable finger length 0.45. Leg I: femur 0.79/0.13 (6.08), patella 0.59/0.13 (4.54), tibia 0.63/0.10 (6.30), basitarsus 0.45/0.10 (4.50), telotarsus 0.43/0.10 (4.30). Leg IV: femur + patella 1.52/0.21 (7.24), tibia 1.22/0.12 (10.17), basitarsus 0.59/0.10 (5.90), telotarsus 0.79/0.10 (7.90).

##### Distribution.

This species is known only from the type locality.

##### Remarks.

*Stenohya huangi* sp. n. is only known from the female, but it can be easily separated from most other species of this genus by the proportions of pedipalpal femur and patella (Table 1). Two species, *Stenohya xiningensis* Zhao et al., 2011 and *Stenohya kashmirensis*,are only known from males, while two others, *Stenohya lindbergi* and *Stenohya vietnamensis* are only known from nymphs. *Stenohya huangi* differs from *Stenohya xiningensis* by the arrangement of trichobothria on the fixed chelal finger: *it* and *et* are at the same level (Fig. 22) in *Stenohya huangi*, whereas in *Stenohya xiningensis* it lies about midway between *est* and *et* (Zhao et al. 2011: fig. 28). *Stenohya huangi* differs from *Stenohya kashmirensis* and *Stenohya lindbergi* in having a lower number of teeth on the movable chelal finger (about 30, versus 70 in *Stenohya kashmirensis* and 78 in *Stenohya lindbergi*). Finally, the new species differs from *Stenohya vietnamensis* in having an epistome.

**Table 1.** Proportions of femur and patella of pedipalps in females of *Stenohya*.

**Species**	**Femur**	**Patella**
*Stenohya caelata*	4.50–4.64	3.63–3.64
*Stenohya chinacavernicola*	5.70	4.75
*Stenohya curvata*	5.00–5.24	3.29–3.33
*Stenohya gruberi*	4.77	3.30
*Stenohya hamata*	4.46–4.51	3.32–3.46
*Stenohya heros*	4.50	3.20
*Stenohya huangi*	6.32	5.31
*Stenohya mahnerti*	4.60	3.00
*Stenohya martensi*	5.00	3.90
*Stenohya pengae*	5.18–5.83	3.83–3.93

In most Neobisiidae the lyrifissures near the trochanteral foramen of the pedipalpal coxa number 3 or 4 (Chamberlin 1931). Having examined the arrangements of lyrifissures in *Stenohya pengae* sp. n., *Stenohya huangi* sp. n., *Stenohya curvata* and *Stenohya xiningensis*, we found all of them have 2 lyrifissures in this position. The males of *Stenohya pengae* sp. n. possess 4–5 lyrifissures behind the foramen and 2–3 dorsal lyrifissures, while females have 3–5 lyrifissures near the foramen and 0–3 dorsally (Fig. 15). Males of *Stenohya curvata* have 7 lyrifissures near the foramen (Zhao et al. 2011: fig. 3) and females have 5, but there are no dorsal lyrifissures. The male of *Stenohya xiningensis* (female unknown) has 6 foramenal lyrifissures and 1 dorsal lyrifissure (Fig. 9).

## Supplementary Material

XML Treatment for Stenohya pengae

XML Treatment for Stenohya huangi
